# Supplementary material for: Stacked antiaromatic porphyrins
Source: Nat Commun. 2016 Nov 30;7:13620. doi: 10.1038/ncomms13620 (PMC5141365; doi:10.1038/ncomms13620)

*** solvent and impurities**

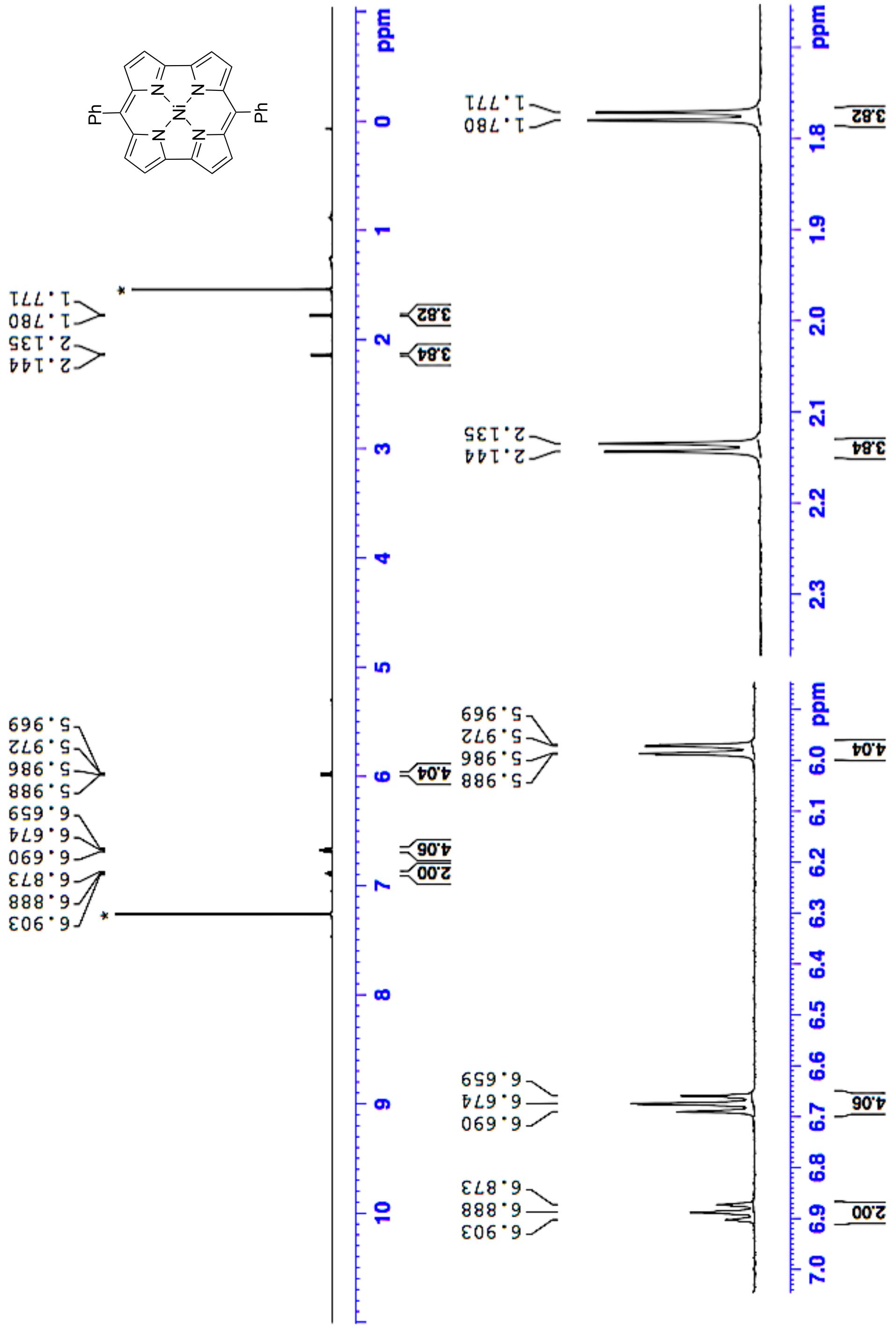

Supplementary Figure 1. ^{1}H NMR spectrum of **3b** in $CDCl_3$ (1.3 mM).

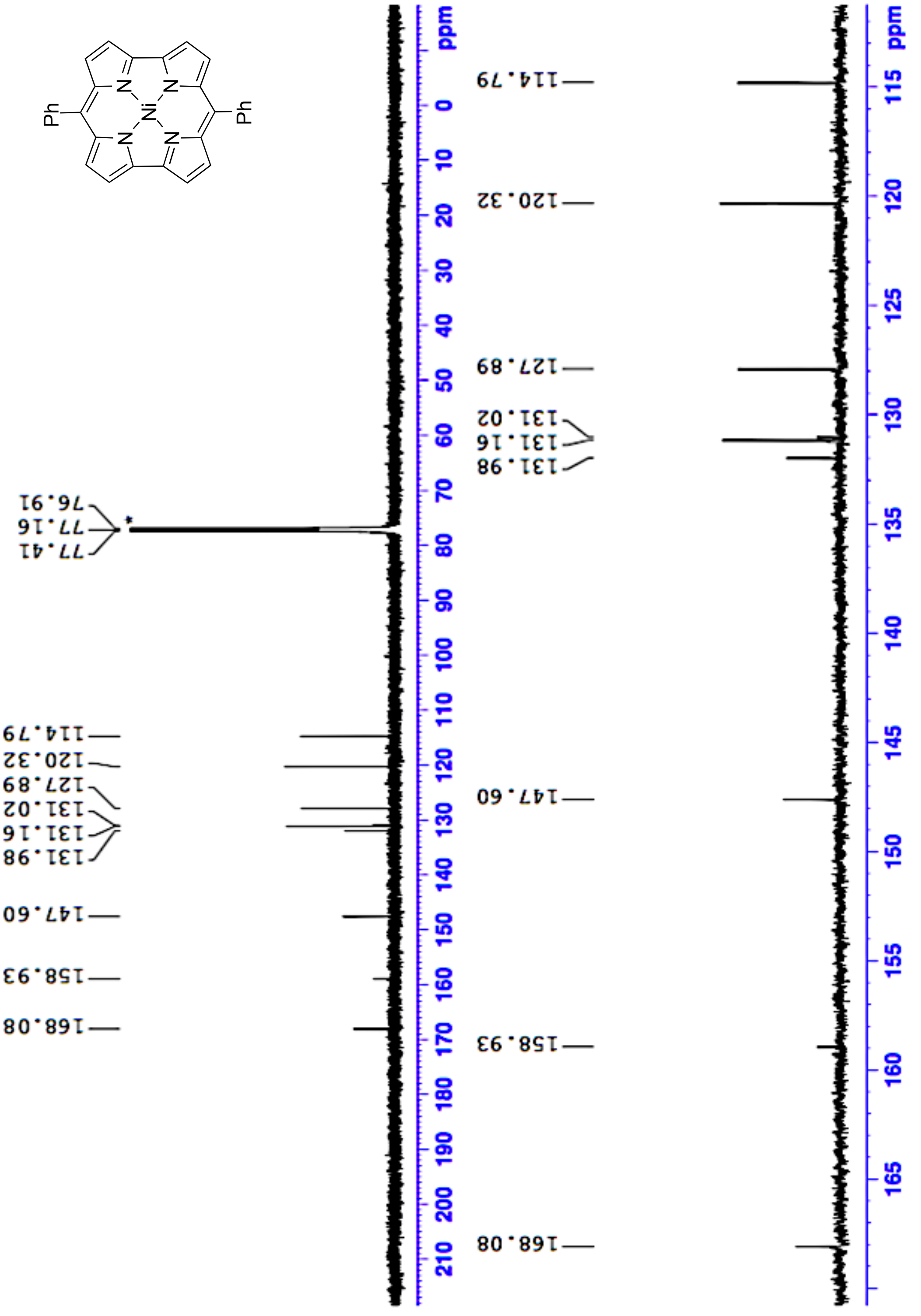

Supplementary Figure 2. ^{13}C NMR spectrum of **3b** in $CDCl_3$ (1.3 mM).

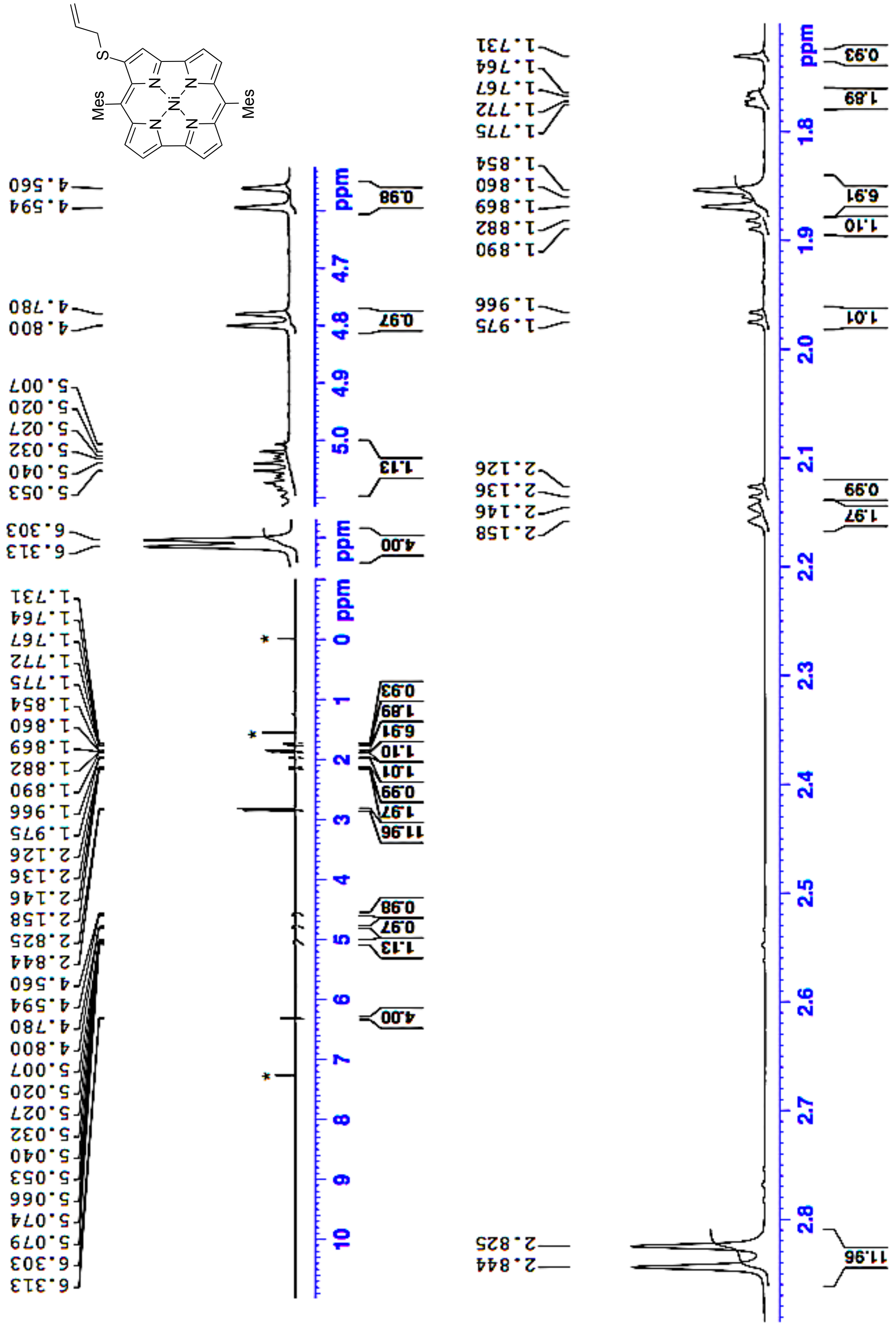

Supplementary Figure 3. ^{1}H NMR spectrum of **4a** in $CDCl_3$.

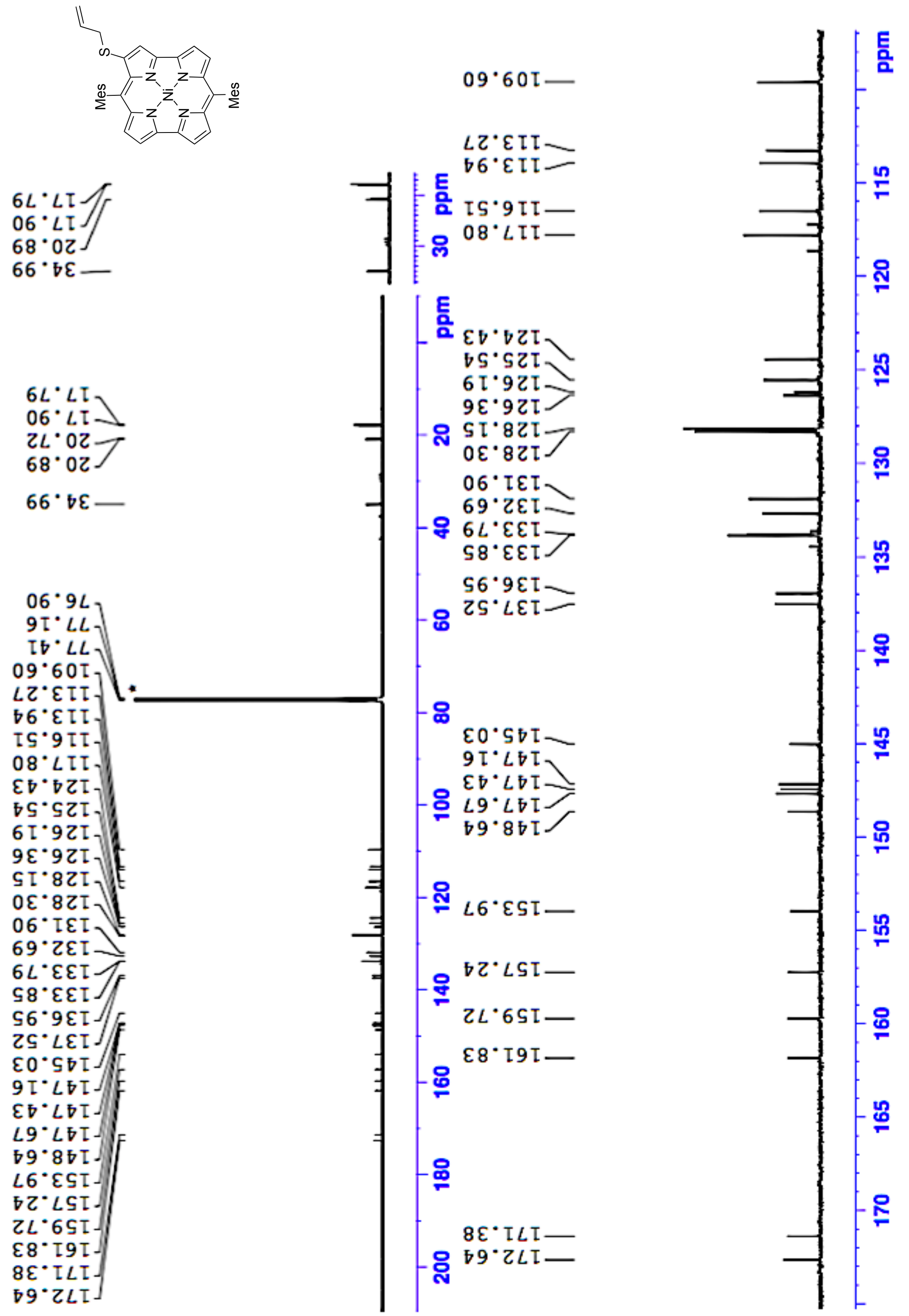

Supplementary Figure 4. ^{13}C NMR spectrum of **4a** in $CDCl_3$.

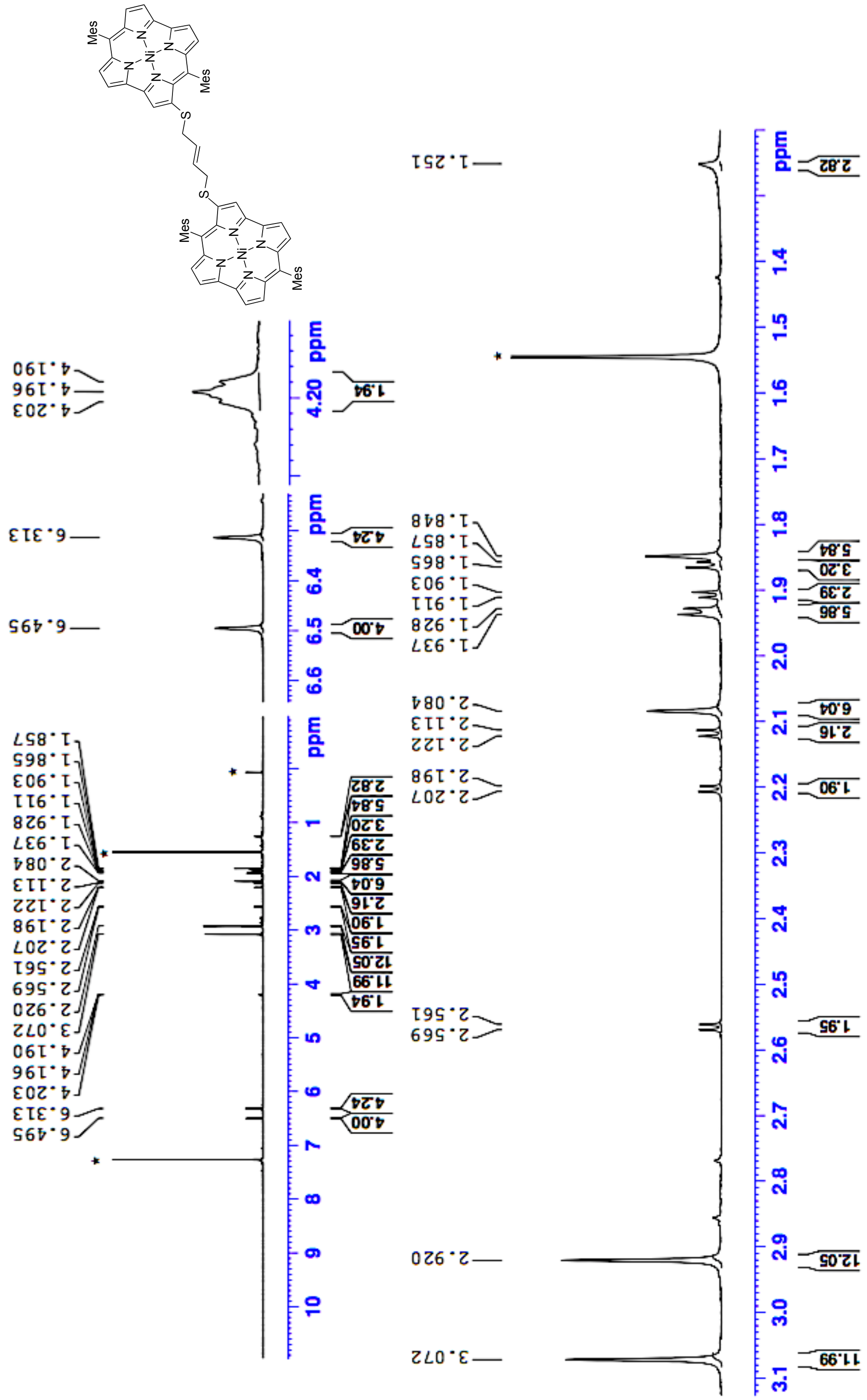

Supplementary Figure 5. ^{1}H NMR spectrum of **5a** in $CDCl_3$.

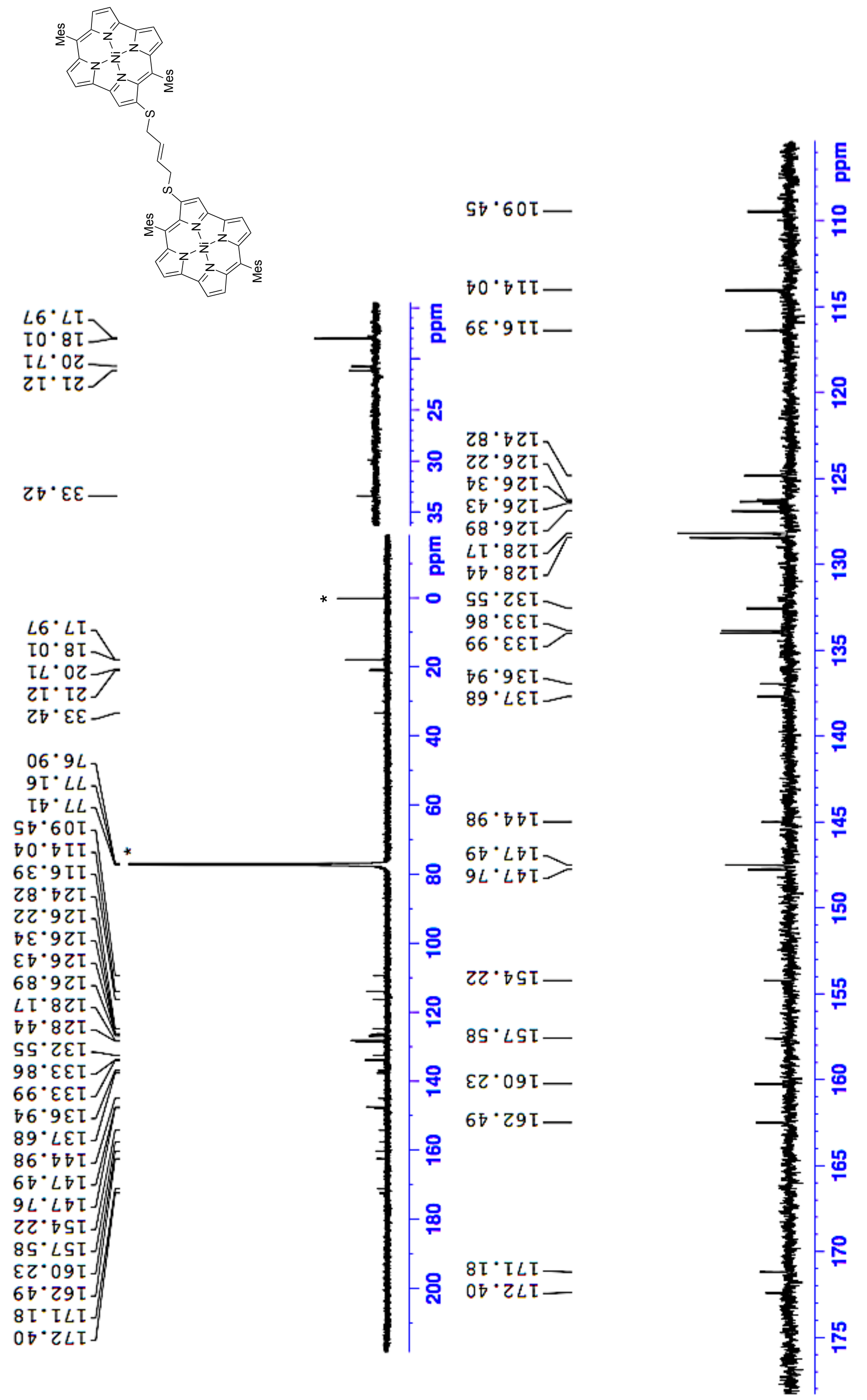

Supplementary Figure 6. ^{13}C NMR spectrum of **5a** in $CDCl_3$.

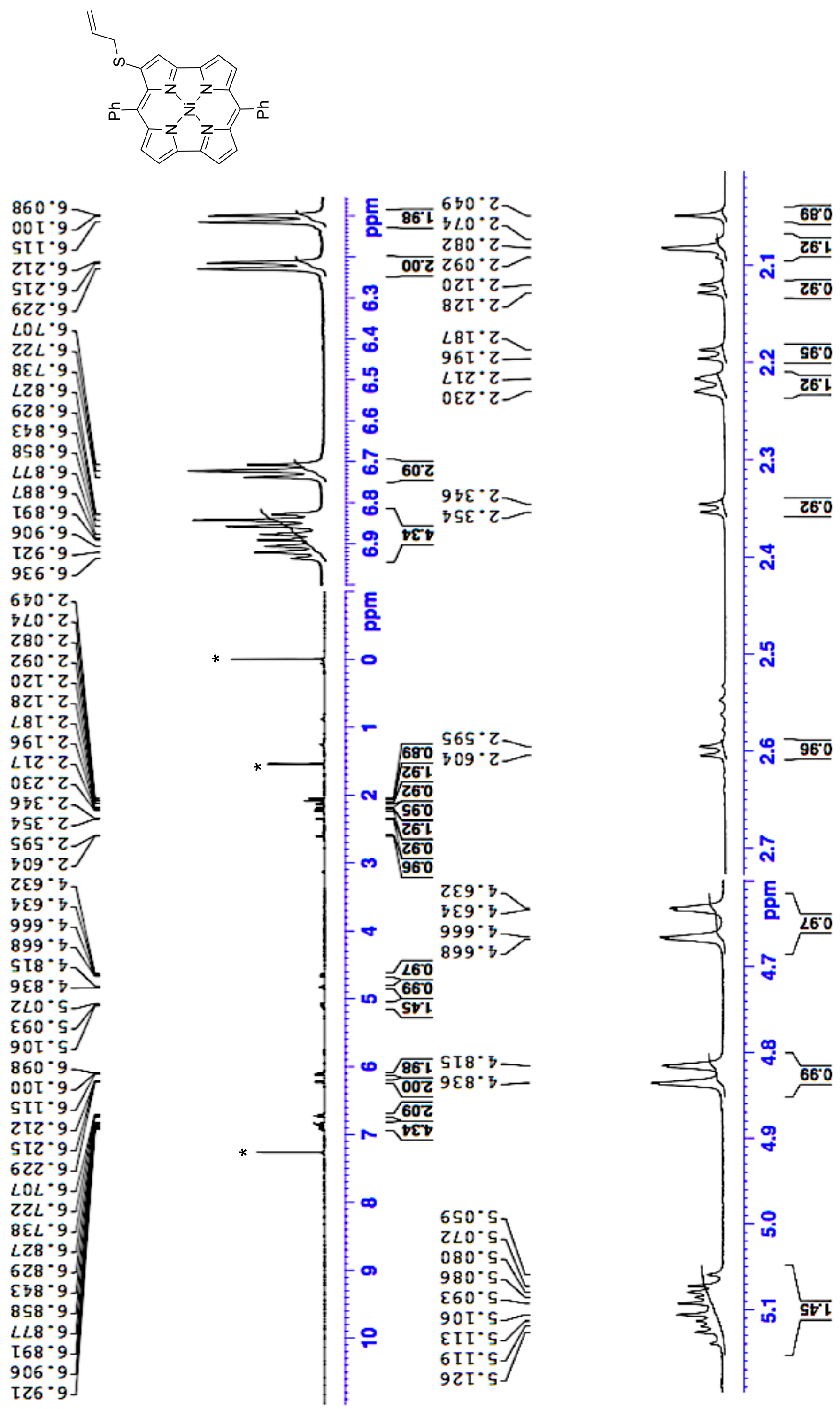

Supplementary Figure 7. ^{1}H NMR spectrum of **4b** in $CDCl_3$.

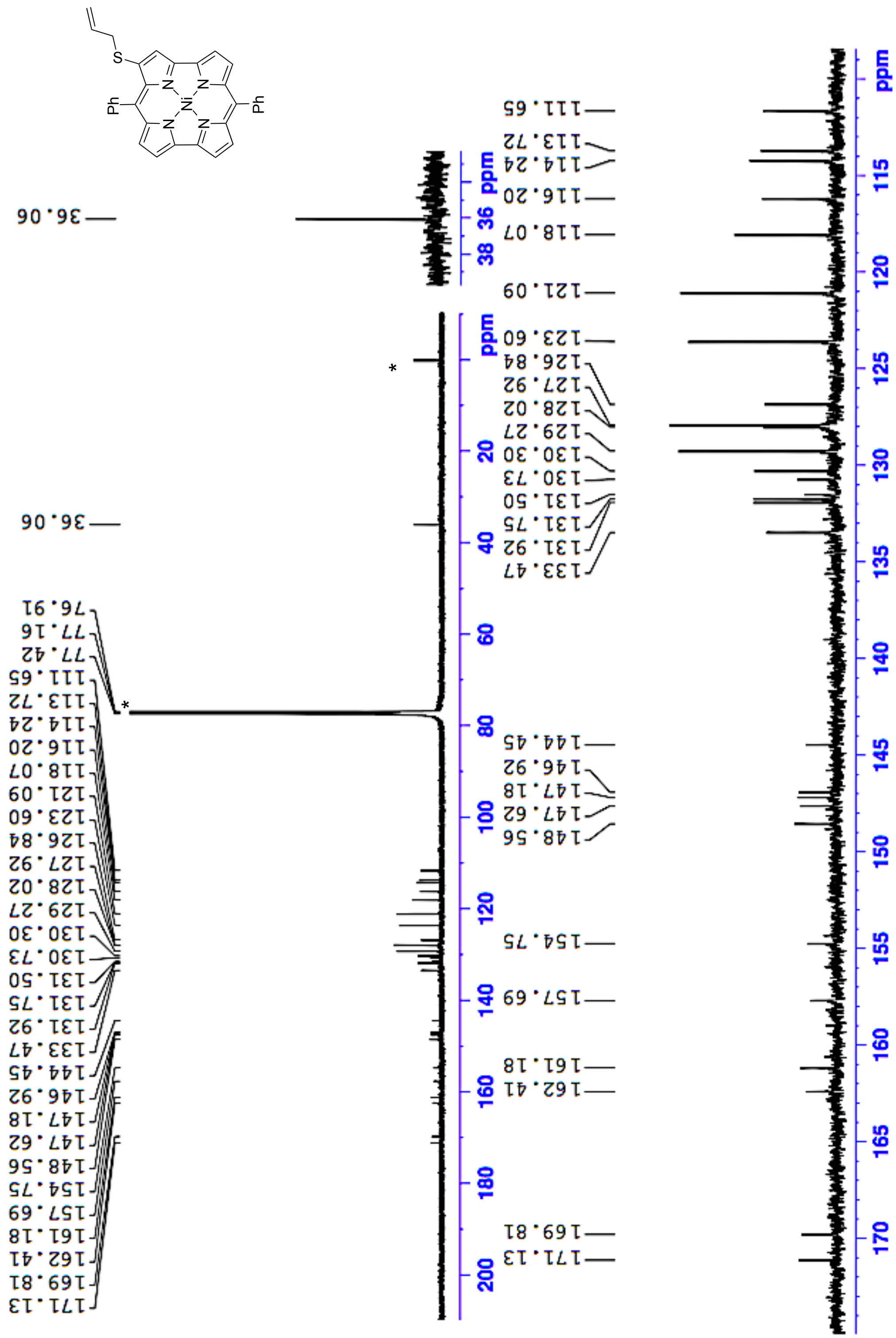

Supplementary Figure 8. ^{13}C NMR spectrum of **4b** in $CDCl_3$.

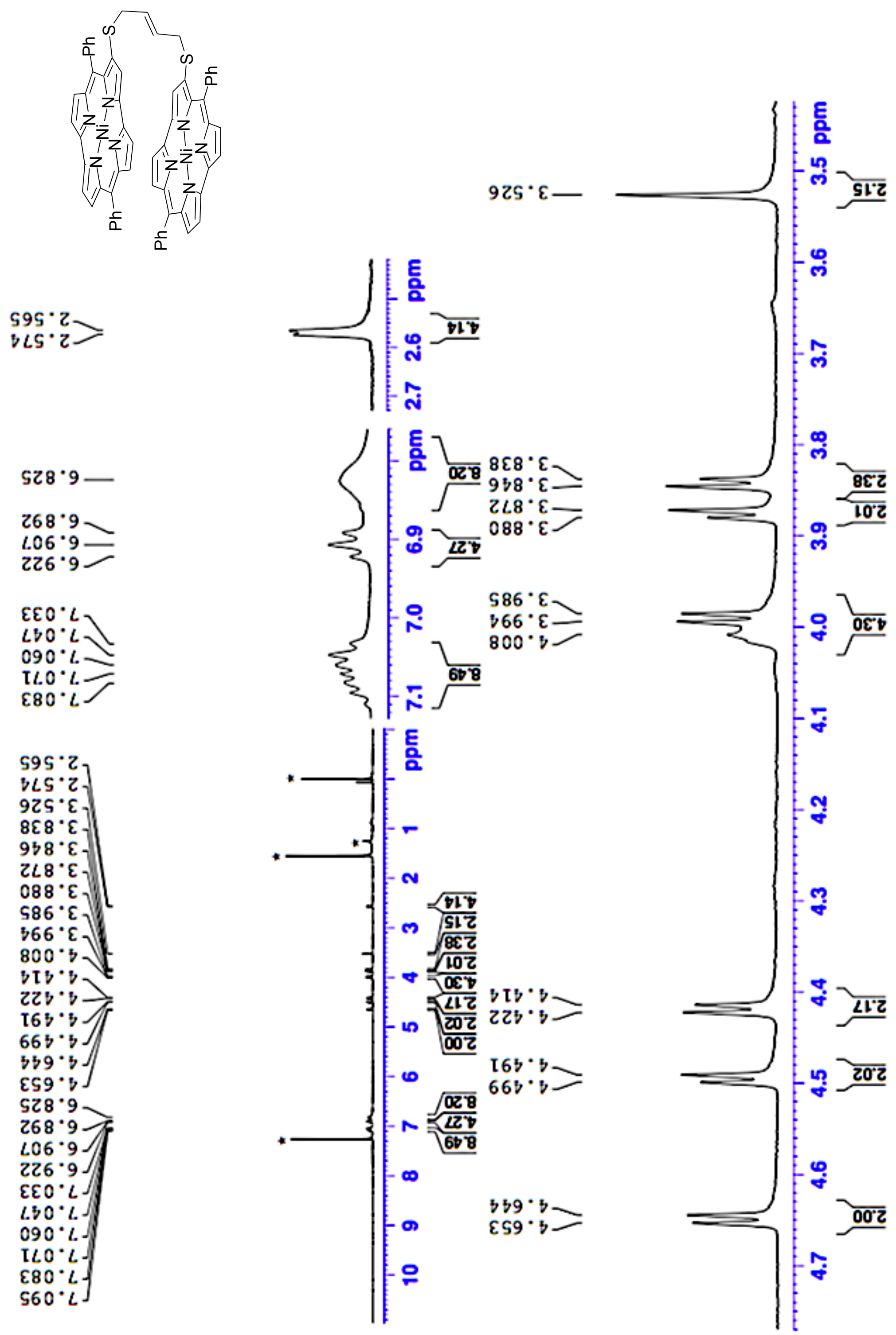

Supplementary Figure 9. ^{1}H NMR spectrum of **5b** in $CDCl_3$.

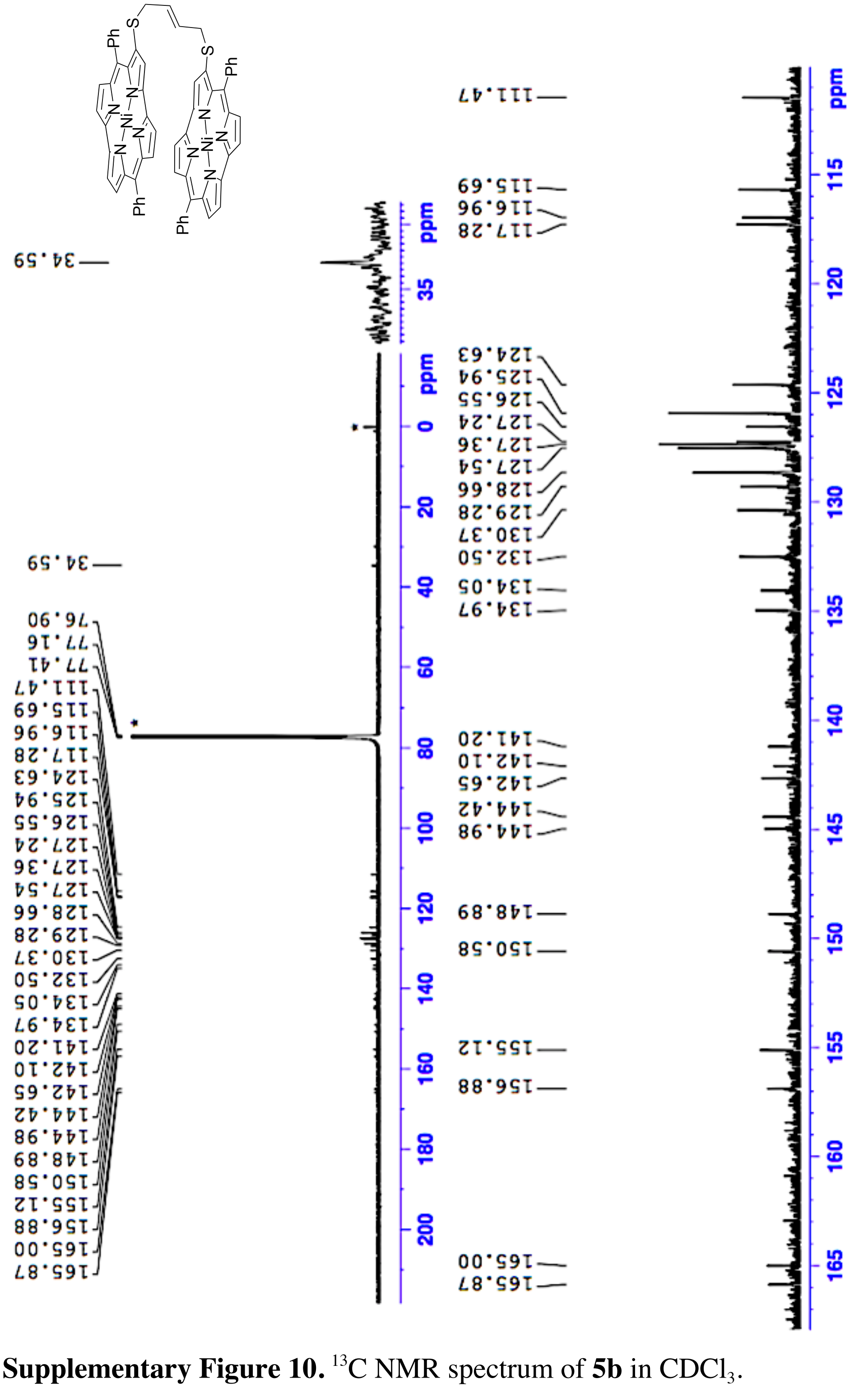

Supplementary Figure 10. ^{13}C NMR spectrum of **5b** in $CDCl_3$.

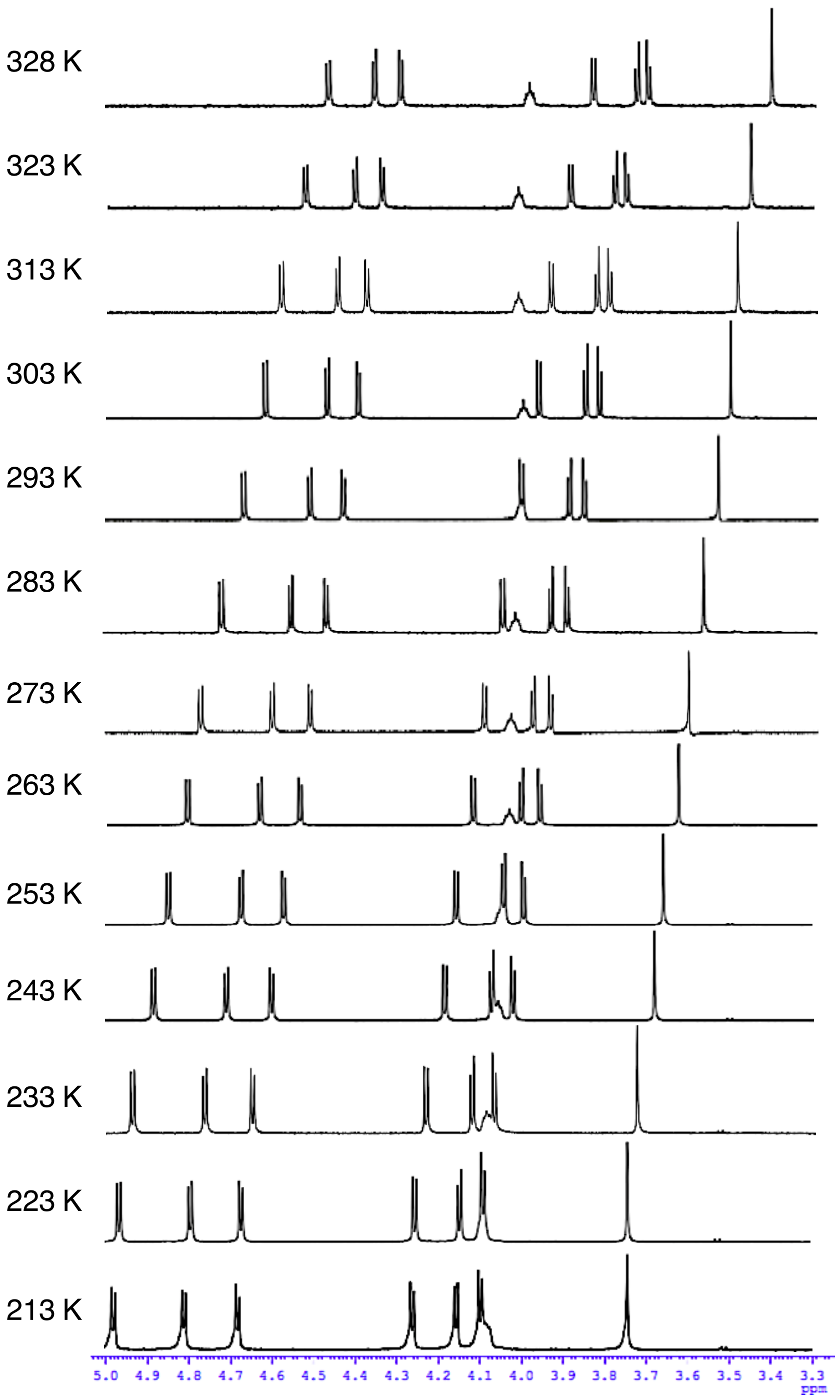

Supplementary Figure 11. Temperature dependence of ^{1}H NMR chemical shifts of **5b** ($CDCl_3$, 213–328 K).

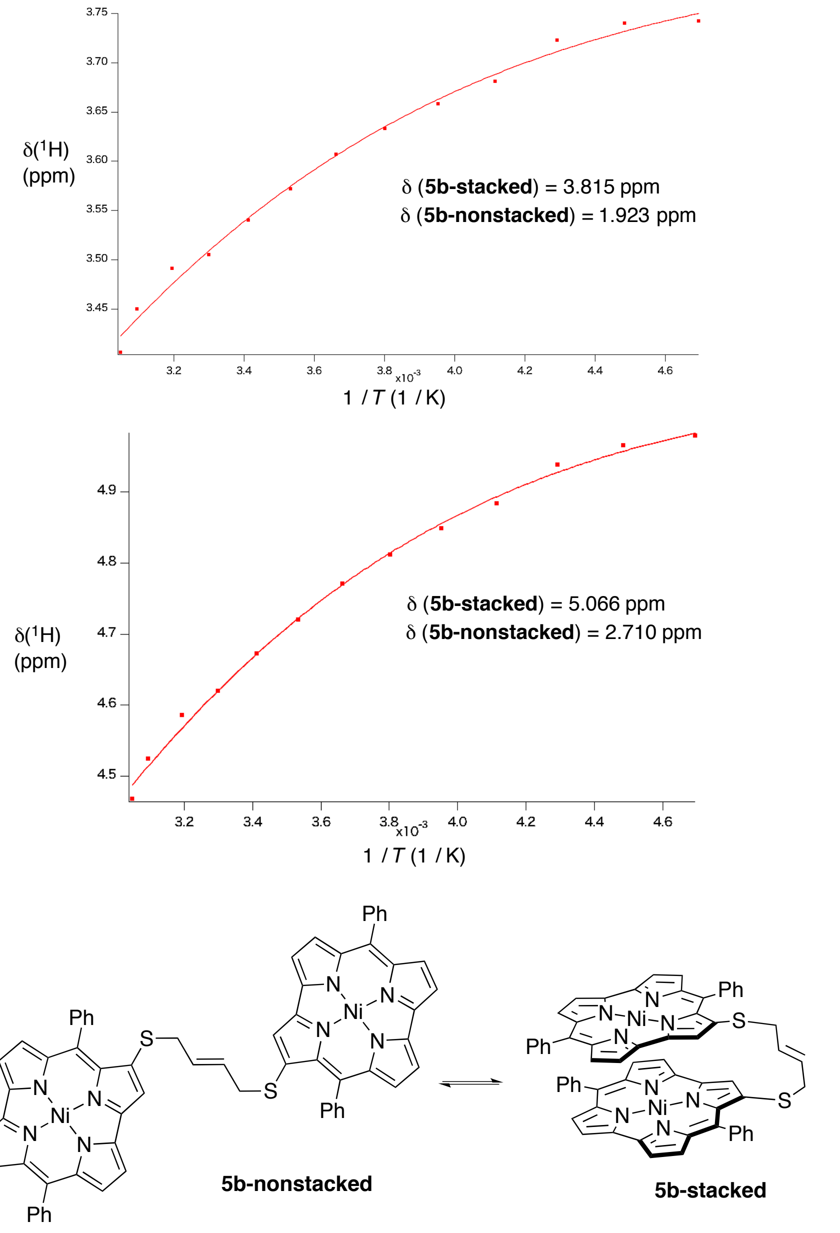

Supplementary Figure 12. ^{1}H NMR chemical shifts of **5b** in $CDCl_3$ plotted as a function of temperature. Filled circles represent experimental points. Solid curves correspond to chemical shift calculated from the van't Hoff equation.

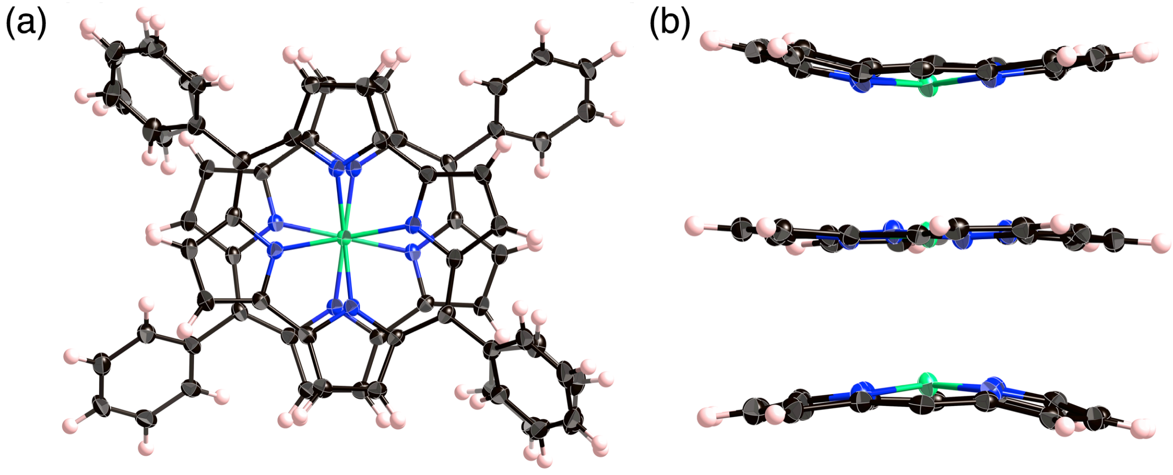

Supplementary Figure 13. X-ray crystal structure of **3b**. (a) Top view and (b) side view. The thermal ellipsoids are scaled at 50% probability level. The phenyl groups are omitted for clarity.

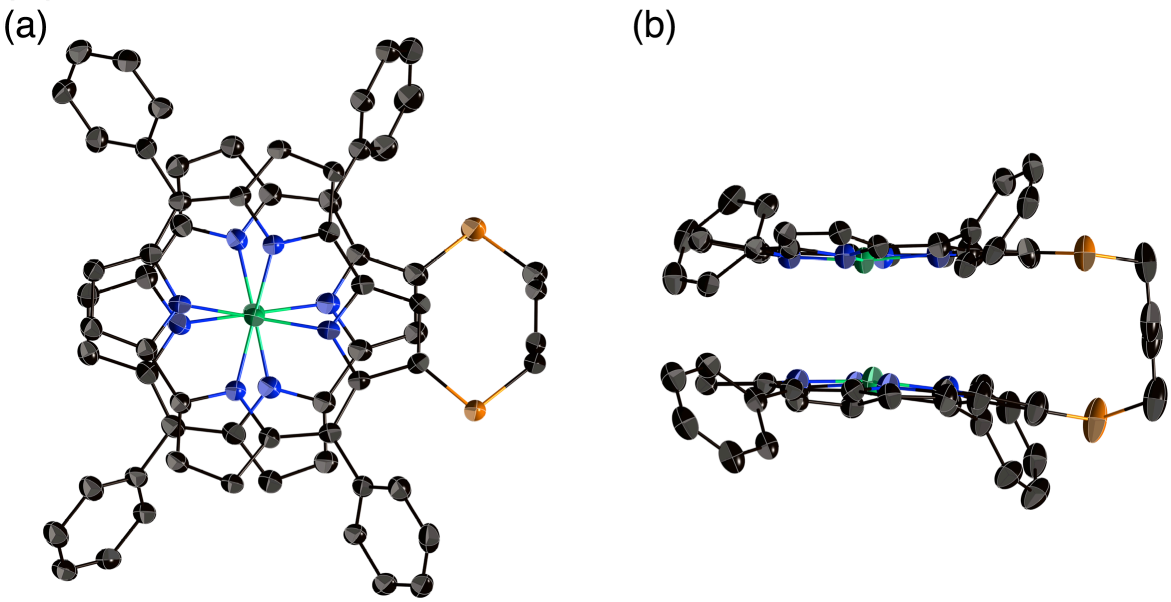

Supplementary Figure 14. X-ray crystal structure of **5b**. (a) Top view and (b) side view. The thermal ellipsoids are scaled at 50% probability level.

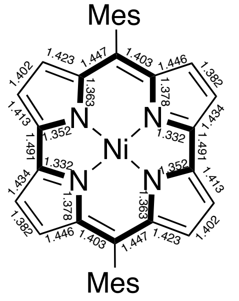

3a
HOMA = 0.45

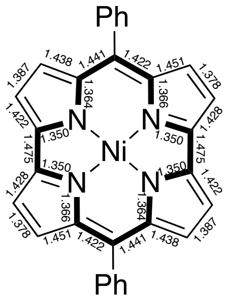

3b (center)
HOMA = 0.56

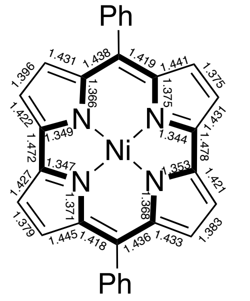

3b (outer)
HOMA = 0.58

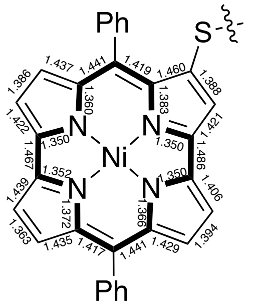

5b
HOMA = 0.54

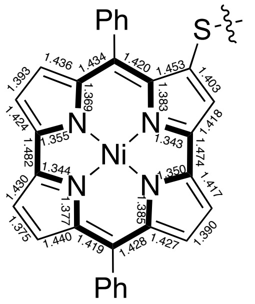

5b
HOMA = 0.56

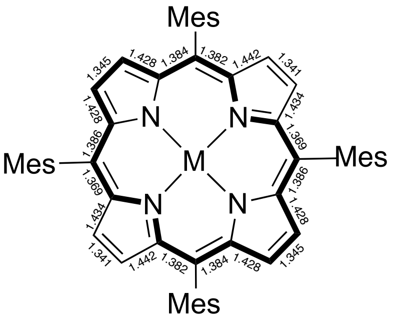

Tetramesitylporphyrin Ni(II)
HOMA = 0.77

Supplementary Figure 15. HOMA values of **3a**, **3b**, **5b** and tetramesitylporphyrin Ni(II) in their X-ray structures. The bonds used for HOMA calculations are indicated in bold lines.

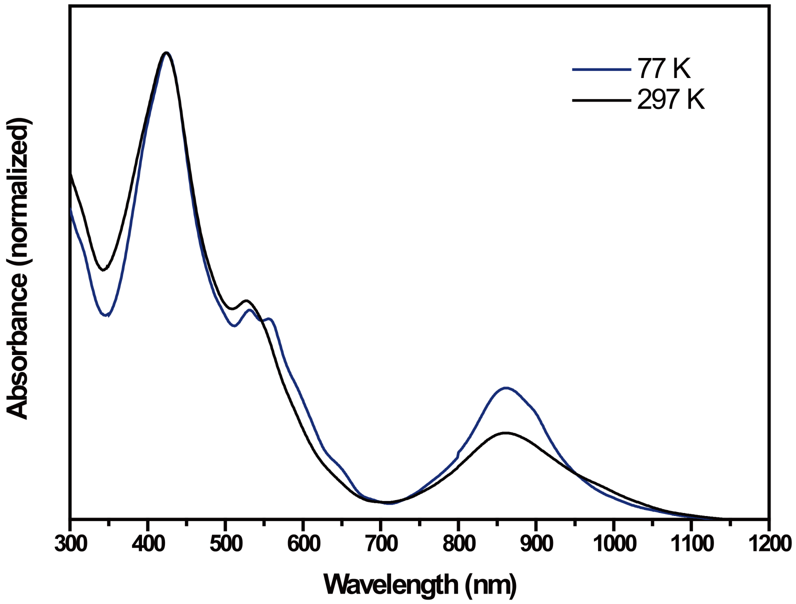

Supplementary Figure 16. Temperature dependent absorption spectra of **5b** at 297 and 77 K in 2-methyl- tetrahydrofuran.

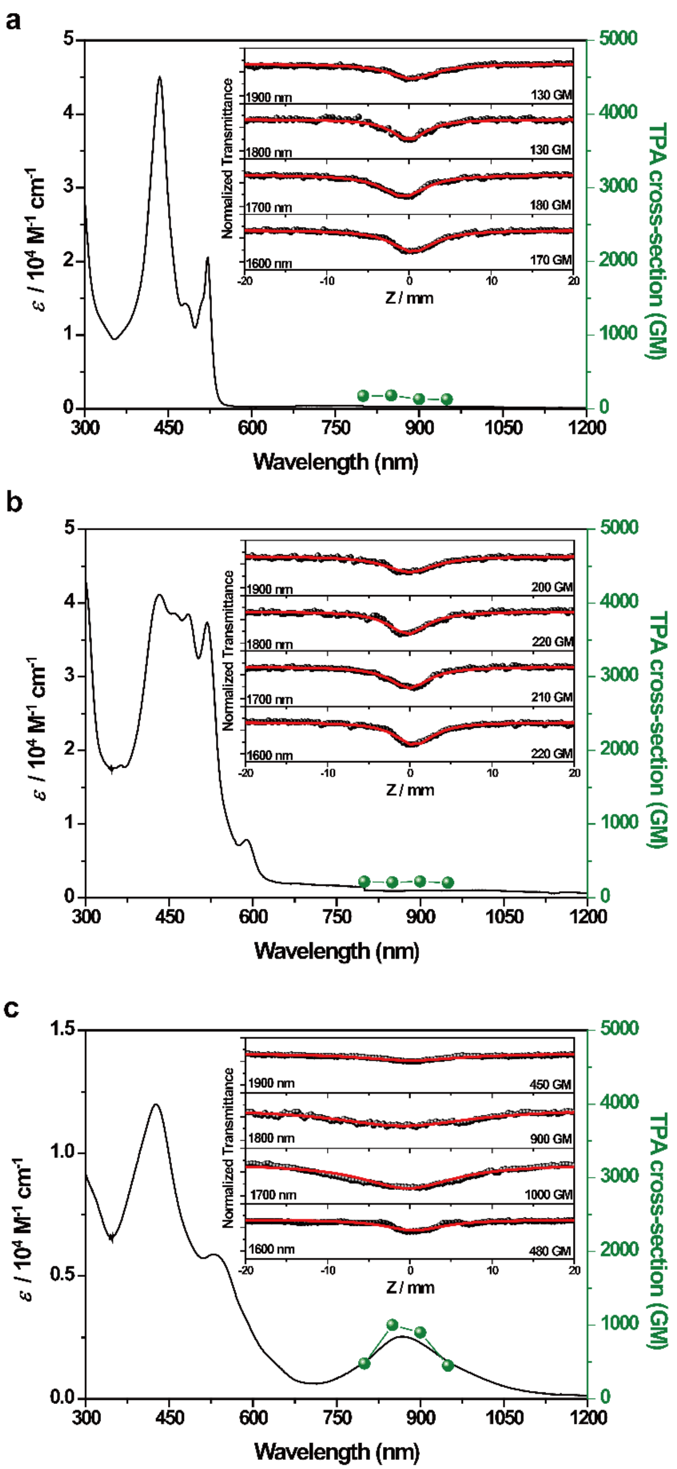

Supplementary Figure 17. Two photon absorption cross-section values of (a) **3a**, (b) **5a** and (c) **5b** measured at 1600, 1700, 1800, and 1900 nm in toluene.

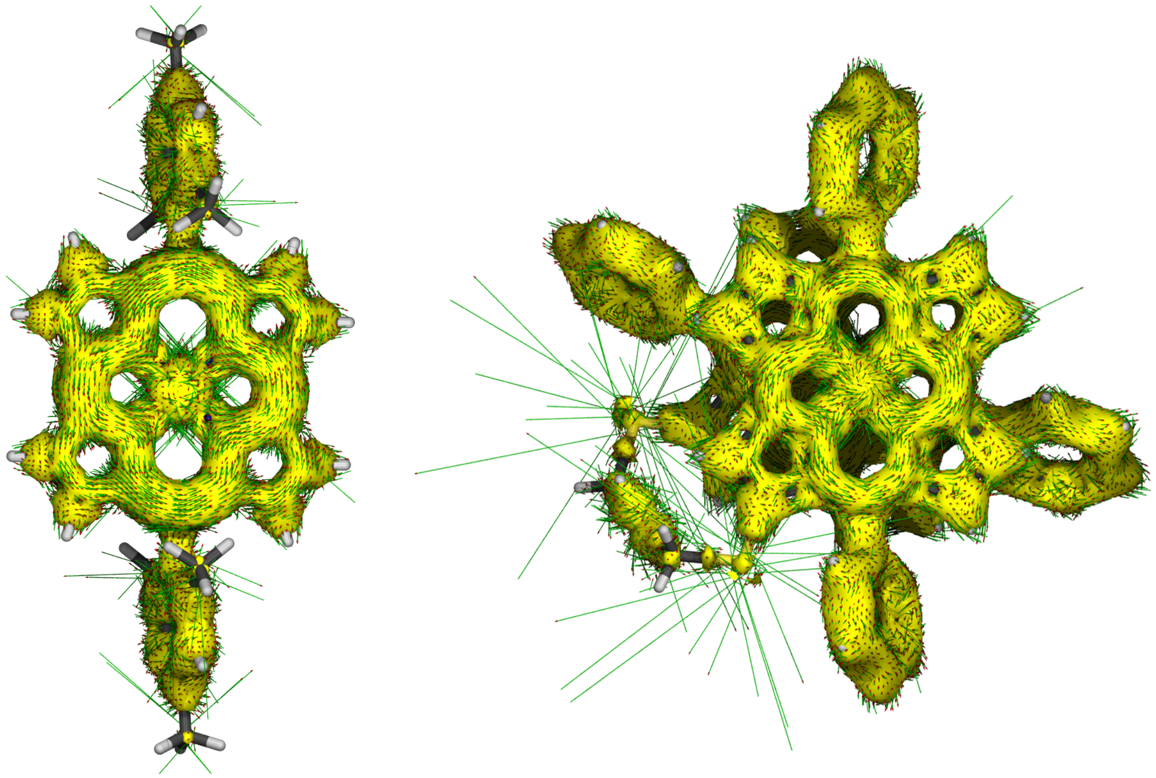

Supplementary Figure 18. ACID plots of **3a** (left) and **5b** (right). Calculations were conducted on the X-ray structures at the CSGT-B3LYP/6-31G(d) level.

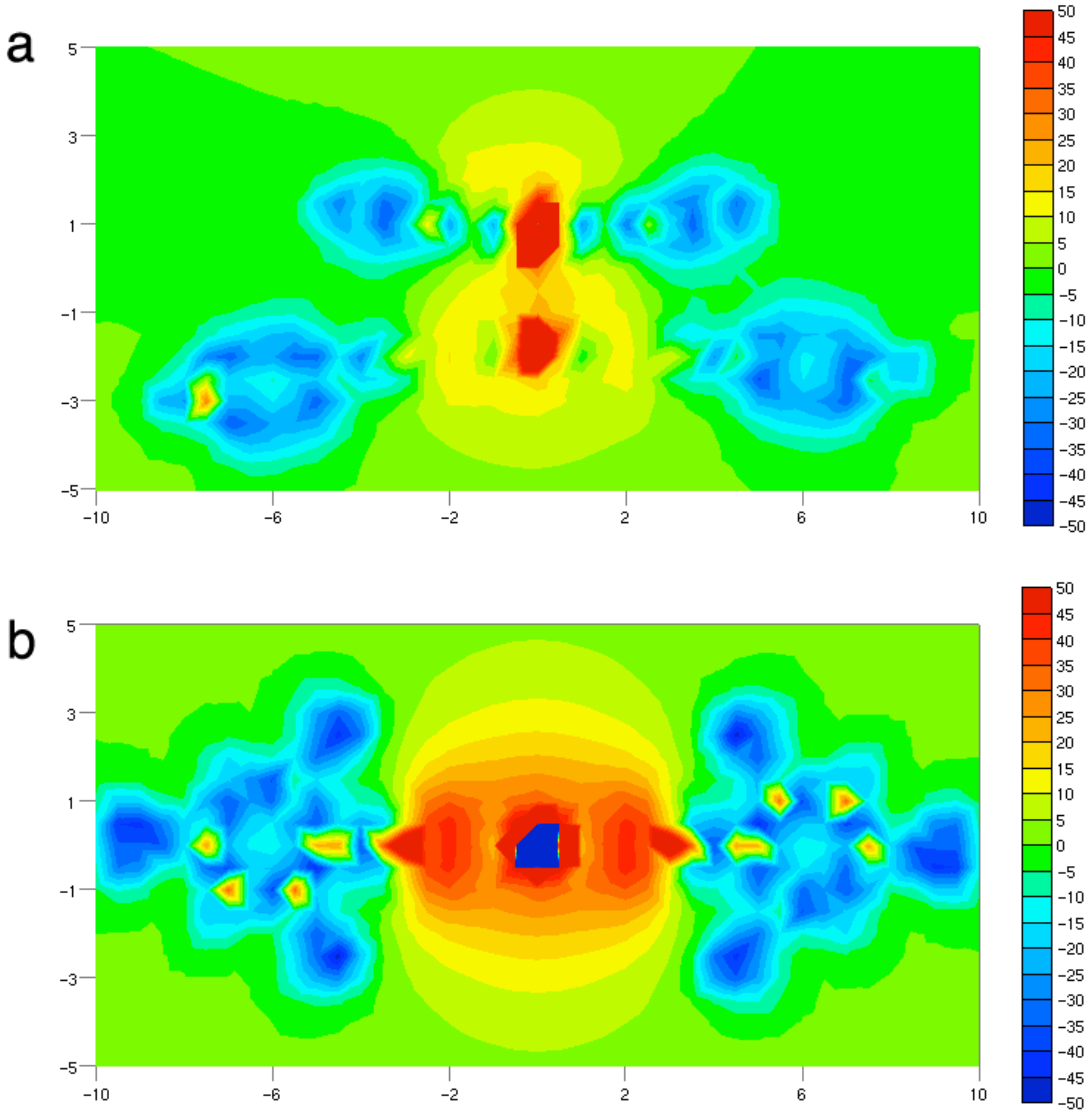

Supplementary Figure 19. NICS plots of **3a** and **5b** at a 0.5 Å grid on the perpendicular plane to the π-systems. Calculations were conducted on the X-ray structures at the GIAO-B3LYP/6-31G(d) level.

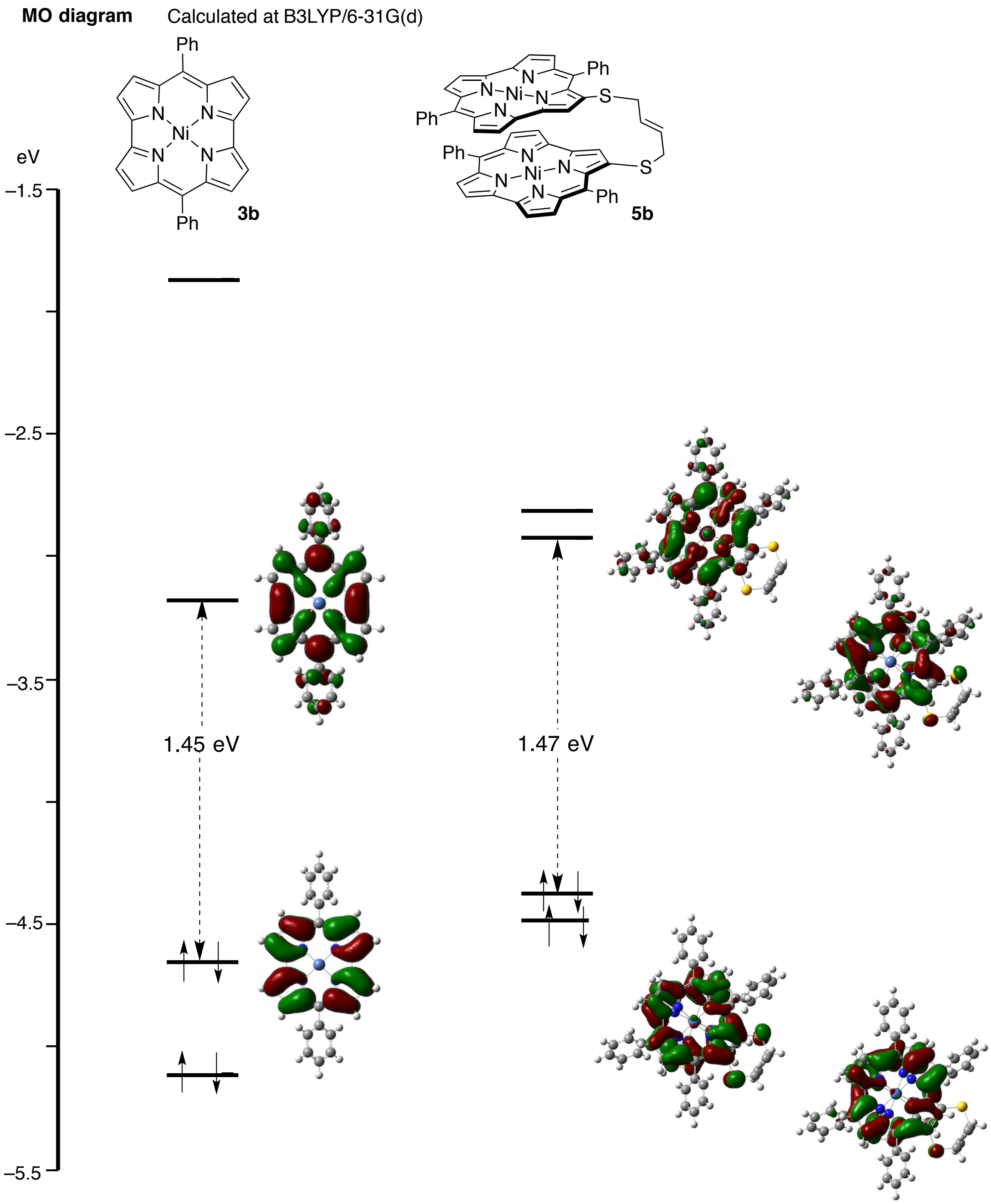

Supplementary Figure 20. Frontier molecular orbitals of **3b** and **5b** at the B3LYP/6-31G(d).

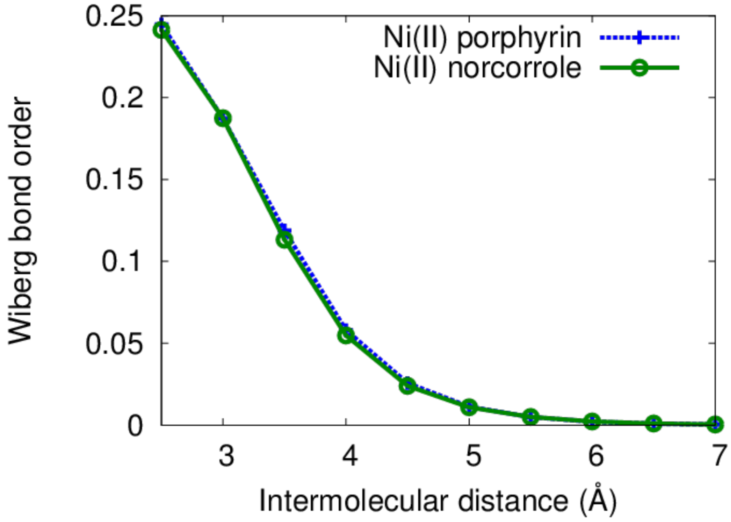

Supplementary Figure 21. Wiberg bond order plots for norcorrole dimer and porphyrin dimer at different stacking distances.

Supplementary Table 1. Summary of Crystallographic data for **3b** and **5b**.

	**3b**	**5b**
empirical formula	$3(C_{30}H_{18}N_4Ni)$	$C_{64}H_{40}N_8S_2Ni_2$, $2(CHCl_3)$
formula weight	1479.58	1341.28
habit	prism	block
T, K	153(2)	93
crystal system	Monoclinic	Monoclinic
space group	$P2_1/c$	$C2/c$
a, Å	13.6420(11)	24.7680(2)
b, Å	11.1624(9)	13.84760(10)
c, Å	21.3653(16)	33.0065(3)
α, deg	90.00	90.0000
β, deg	95.271(2)	99.5763(4)
γ, deg	90.00	90.0000
V, Å^3	3239.7(4)	11162.73(16)
Z	2	8
Dc, g/cm^3	1.517	1.596
$F(000)$	1524	2276.00
crystal size, mm^3	0.40 × 0.40 × 0.20	0.20 × 0.05 × 0.02
$2\theta_{max}$, °	56.62	58.00
R_{int}	0.036	0.077
R_1 ($I > 2\sigma(I)$)	0.0508	0.0719
wR_2 (all data)	0.1386	0.2038
GOF	1.034	1.067
observed reflections	6291	9694
No. of unique reflections	8037	10347
No. of reflections measured	22640	68849
parameters	475	793

Supplementary Table 2. Character and second–order interaction energies of strongly interacting donor–acceptor NBOs in norcorrole dimer and porphyrin dimer. The stacking distance was fixed to be 3.0 Å. Donor–acceptor NBO interactions between a) two stacked norcorroles and b) two stacked porphyrin are shown below.

Compound	Pair label	Donor NBO	Acceptor NBO	Energy (kcal mol^{-1})
Norcorrole dimer	(i)	lone pair on C	π* C=N	6.33
	(ii)	π* C=N	π* C=C	3.89
	(iii)	lone pair on C	π* C=N	3.82
Porphyrin dimer	(i)	π* C=N	π* C=C	2.72
	(ii)	π* C=N	π* C=C	2.70
	(iii)	π* C=N	π* C=C	2.68

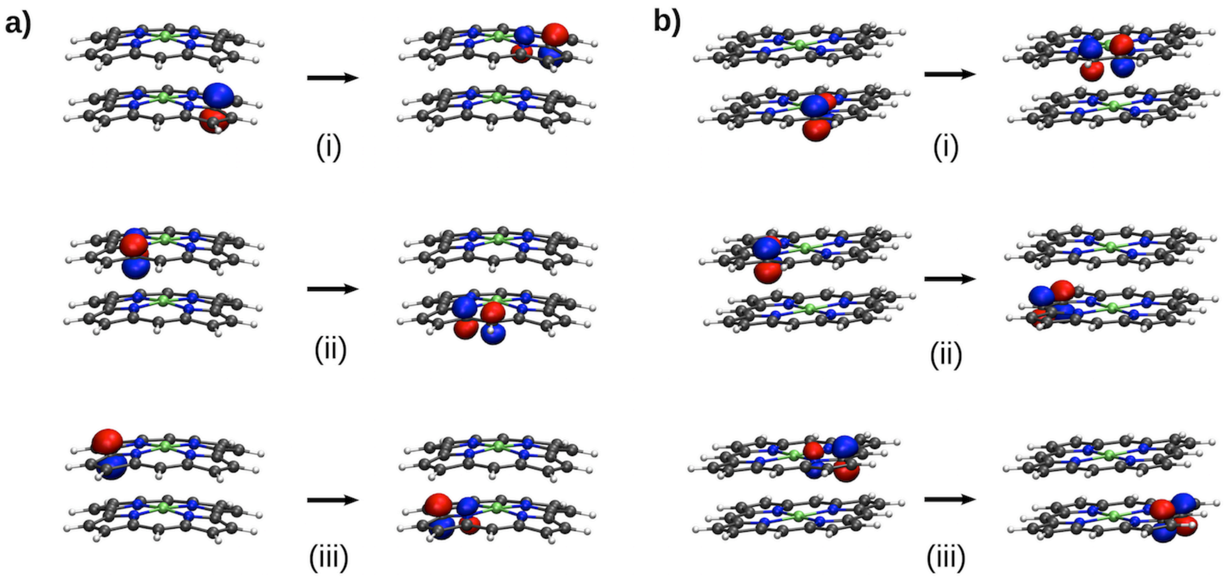

Supplement: Supplementary Information — Supplementary figures 1-21 and supplementary tables 1-2. [file ncomms13620-s1.pdf]
